# Mechanical Performances Analysis and Prediction of Short Plant Fiber-Reinforced PLA Composites

**DOI:** 10.3390/polym15153222

**Published:** 2023-07-28

**Authors:** Wenlong Mu, Xianglin Chen, Shijie Li, Yufeng Sun, Qingpeng Wang, Jingxin Na

**Affiliations:** 1 College of Mechanical and Electrical Engineering, Henan Agricultural University, Zhengzhou 450002, China; 2 State Key Laboratory of Automotive Simulation and Control, Jilin University, Changchun 130022, China

**Keywords:** short plant fiber, PLA, surface treatments, mechanical properties, performance prediction

## Abstract

Plant fiber-reinforced polylactic acid (PLA) exhibits excellent mechanical properties and environmental friendliness and, therefore, has a wide range of applications. This study investigated the mechanical properties of three short plant fiber-reinforced PLA composites (flax, jute, and ramie) using mechanical testing and material characterization techniques (SEM, FTIR, and DSC). Additionally, we propose a methodology for predicting the mechanical properties of high-content short plant fiber-reinforced composite materials. Results indicate that flax fibers provide the optimal reinforcement effect due to differences in fiber composition and microstructure. Surface pretreatment of the fibers using alkali and silane coupling agents increases the fiber–matrix interface contact area, improves interface performance, and effectively enhances the mechanical properties of the composite. The mechanical properties of the composites increase with increasing fiber content, reaching the highest value at 40%, which is 38.79% higher than pure PLA. However, further increases in content lead to fiber agglomeration and decreased composite properties. When the content is relatively low (10%), the mechanical properties are degraded because of internal defects in the material, which is 40.42% lower than pure PLA. Through Micro-CT technology, the fiber was reconstructed, and it was found that the fiber was distributed mainly along the direction of injection molding, and the twin-screw process changes the shape and length of the fiber. By introducing the fiber agglomeration factor function and correcting the Halpin-Tsai criterion, the mechanical properties of composite materials with different contents were successfully predicted. Considering the complex stress state of composite materials in actual service processes, a numerical simulation method was established based on transversely isotropic material using the finite element method combined with theoretical analysis. The mechanical properties of high-content short plant fiber-reinforced composite materials were successfully predicted, and the simulation results showed strong agreement with the experimental results.

## 1. Introduction

As human societies and economies continue to develop rapidly and transportation infrastructure expands, the consumption of oil energy, a typical non-renewable fossil energy source, is being depleted at an alarming rate. This phenomenon has led to the inevitable problems of environmental pollution and resource shortages. Nowadays, countries worldwide are dedicated to the research and development of new renewable energy sources, with biodegradable materials being a hot research topic in recent years. One typical biodegradable material is PLA, which comes from a diverse range of sources, often using starch extracted from renewable crops as the synthetic raw material [1,2]. PLA products have the remarkable ability to completely degrade into CO_2_ and H_2_O under composting conditions after disposal. This process enables the products to re-enter the ecological cycle, effectively avoiding pollution problems associated with the use of petroleum-based plastics. Additionally, PLA products offer several advantages, such as good biocompatibility, ease of processing, and low energy consumption during production [3]. Despite its many advantages, PLA has certain limitations, including high brittleness, poor thermal stability, and weak mechanical properties, which restrict its widespread application and promotion. To overcome these challenges, it is often necessary to modify pure PLA by mixing it with appropriate enhancement phases [4]. Currently, scholars primarily use plant fibers and synthetic fibers to reinforce polylactic acid (PLA) in order to overcome its weaknesses [5,6]. Compared to synthetic fibers, plant fibers have the advantages of convenience, low cost, high specific strength, renewability, and biodegradability [7]. Plant fiber-reinforced PLA composites (PFRP), which align with the current trend of environmental protection and energy conservation, have become a research hotspot in the field of green composites [7,8,9].

The mechanical performances of plant fiber-reinforced composites are affected by many factors, including manufacturing processes, fiber type, pretreatment processes, and fiber content [10,11,12]. The preparation process of PFRP depends on various factors, such as composite size and fiber length. Common methods for preparing PFRP include injection molding, extrusion injection molding, extrusion compression molding, hot pressing, film stacking, pultrusion, and additive manufacturing (3D printing) [5]. Le et al. [13] used 3D printing to prepare continuous flax fiber-reinforced polylactic acid composites, and Oksman et al. [14] used a twin-screw processing technique to prepare polylactic acid composites reinforced with different contents of flax fibers. Commonly used plant fibers for PFRP composites include wood fiber, hemp fiber, straw fiber, and bamboo fiber. However, the mechanical behavior of PFRP composites may be inconsistent due to variations in plant fiber components and their respective mechanical properties [15,16,17,18]. Generally, the mechanical properties of composites increase with the increase in fiber load-bearing capacity [19]. In a study by Bajpai et al. [20], the tensile strength of nettle and sisal fiber-reinforced PLA composites was compared with that of pure PLA. The results showed that the addition of fibers improved the tensile properties of the material, and the mechanical properties of the fibers themselves greatly differed, leading to a significant impact of fiber type on the mechanical properties of the PFRP composites. 

For fiber-reinforced composites, in addition to the performances of the fibers and the matrix, the interfacial bonding ability between the fibers and the matrix is also important in determining the mechanical properties of the composite [7]. Like synthetic fiber-reinforced composites, there are two primary methods to enhance the properties of the plant fiber–matrix interface in PFRP composites: chemical treatment and physical treatment. Physical treatments involve methods such as corona, plasma, UV, and heat treatment, while chemical treatments mainly include alkali soaking, acetylation, and silane treatments [15]. Compared with physical treatment, chemical treatment is cheaper and more convenient, so it has gained wide attention in engineering applications and preparation technology research [21]. Alkali treatment effectively removes hemicellulose, lignin, pectin, and waxes from plant fibers, resulting in an increased roughness of the fiber surface and improved interfacial bonding performance in PFRP [22]. The silane coupling agent contains different functional groups at both ends and can serve as a bridge between the fiber and the matrix. The agent connects to the hydrophilic group of the fiber at one end and the hydrophobic group in the matrix at the other end, thereby enhancing the interfacial bonding and improving the mechanical properties of the composite [15]. Furthermore, the properties of composites treated with both alkali and silane were further improved [23]. Due to the greater mechanical properties of plant fibers compared to PLA matrix, the strength and stiffness of composites typically increase with increasing fiber content. However, beyond a certain percentage of fiber content, a decrease in material properties may occur due to a decrease in fiber–matrix interfacial stress and an increase in porosity [24]. Le et al. [25] examined the impact of fiber content on the mechanical properties of continuous unidirectional flax fiber-reinforced epoxy resins. The study revealed that the tensile strength and Young’s modulus of the material improved with increased fiber content, but when the content reached 63%, the tensile strength decreased, and the Young’s modulus no longer changed.

Experimental studies can characterize the mechanical properties of plant fiber-reinforced composites accurately. However, there are numerous factors that affect the mechanical properties of plant fiber composites, and multiple experiments are accompanied by additional time and cost. Theoretical models have been proposed by current researchers to simulate and analyze the properties of plant fiber composites. These models include the Halpin–Tsai theory, Hirsch theory, Tsai–Wu theory, and modified Bowyer–Bader theory [26]. Although theoretical models are useful for simplifying the material design process, they cannot fully reflect the influence of various factors. Therefore, the simulation and prediction of mechanical properties of composite structures based on the finite element method (FEM) is an effective alternative to expensive experimental tests. This approach can significantly improve the efficiency of performance design [27,28,29]. Most of the existing studies on the simulation of plant fiber composites mainly focus on continuous unidirectional fiber-reinforced composites. Zhong et al. [28] utilized a multi-scale representative volume element method to accurately predict the mechanical properties of continuous unidirectional flax/polypropylene composites. Usually, accurately modeling and simulating the performance of high-content short plant fiber-reinforced composites is more difficult due to the large aspect ratio and randomly distributed fibers. Kari et al. [29] achieved performance prediction of randomly distributed short-fiber composites with 25% content by adjusting the fiber aspect ratio to 1:1. The common modeling and performance prediction methods for short-fiber composites are suitable for low-volume content (less than 5%), studies have shown that plant fibers require higher contents (20% or more) to provide significant enhancement [30,31]. Therefore, there is currently a lack of accurate and efficient methods to predict the mechanical properties of high content short-fiber-reinforced composites.

Nowadays, there is still a lack of systematic and comprehensive understanding of the effects of different short plant fiber types, pretreatment processes, content, and other design parameters on the performance of PLA composites. Furthermore, simple and effective methods for predicting the performance of high-content short plant fiber composites are limited, which hinders the further application and promotion of PFRP. In this study, three different plant fibers (flax, ramie, and jute) were used to reinforce PLA. The fibers were treated with alkali and silane coupling agents, and short PFRP with varying fiber contents was prepared using a twin-screw extruder. The effects of different short plant fiber types, pretreatment processes, and content on the properties of PLA composites were systematically investigated through mechanical testing and material characterization techniques, such as SEM, FTIR, and DSC. Moreover, we used Micro-CT technology to reconstruct the composite and predicted its mechanical properties using the modified Halpin–Tsai rule. Considering the complex stress state of composites in the actual service process, a numerical simulation method was established based on transversely isotropic using the finite element method combined with theoretical analysis.

## 2. Experimental

### 2.1. Materials

The flax fiber used in this study was sourced from Easy Composites, Beijing, China, while the ramie and jute fibers were obtained from Shanghai Zhang dan Garment Accessories Company, Shanghai, China. PLA pellets (4032D) were supplied by Nature Works, Minneapolis, MN, USA, with a density of 1.24 g/cm^3^ and a melt flow rate index of 7.0 g/10 min (210 °C). Table 1 shows the mechanical property parameters of the three fibers and PLA, including density ρ, tensile strength S, Young’s modulus E, and elongation at break ε (provided by the supplier).

### 2.2. Sample Preparation

The plant fibers were cut into short fibers with lengths of 3–5 mm, washed with distilled water to remove most of the ash and impurities, and then dried in a dryer at 60 °C for 8 h. Once the fibers were completely dried, they were immersed in a 3% NaOH solution for 30 min. An appropriate amount of acetic acid solution was added to neutralize the fibers, and the neutralized fibers were then washed with distilled water. Subsequently, the fibers were dried in a dryer at 40 °C for 36 h. This lower temperature was used because alkali-treated fibers contain a large amount of water, and a lower temperature ensures the integrity of the fibers and avoids cracks caused by the rapid evaporation of water.

A silane coupling agent with a 5% fiber mass fraction was hydrolyzed in an ethanol solution (ethanol/distilled water = 4/1) for 1 h. The alkali-treated fibers were then soaked in the hydrolyzed ethanol solution for another hour and subsequently dried in a dryer at 60 °C for 8 h.

**Figure 1:**
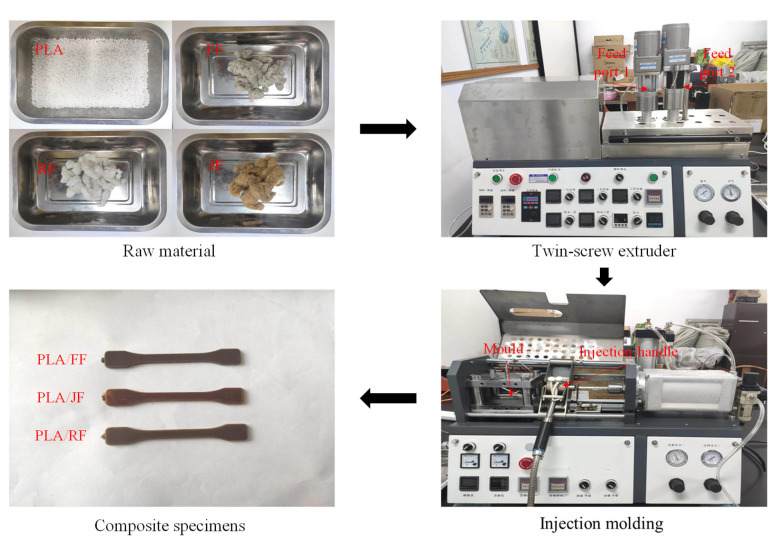
Composite material preparation process.

The plant fiber and PLA pellets were put into the two feed ports of the twin-screw extruder (Wuhan Ruiming Experimental Instrument SJZS-10B, Wuhan, China), respectively. The feed speed was set according to the different contents, and the screw speed was set to 40 rpm. The twin-screw extruder used in this study has two biconical co-rotating screws with an L/D of 250/10 and 250/30, respectively. There are three temperature gradients from the feed zone to the die exit, which are 165 °C in the mixing zone, 175 °C in the reacting zone, and 170 °C at the die exit, respectively. The temperature of the injection handle was set to 175 °C, and the mold was set to 50 °C of the injection molding machine (Wuhan Ruiming Experimental Instrument SZS-20, Wuhan, China) in this study. The extruded filamentary composites were cut off and heated into the injection molding machine injection handle for 10 min. Finally, the composite samples were prepared by injection molding, in which the injection pressure was 55 Bar, and the injection time and holding time were 5 and 15 s, respectively. The preparation process is shown in Figure 1.

### 2.3. Performance Testing

#### 2.3.1. Mechanical Test

The tensile strength of the samples was tested according to ASTM D638 standard using a tensile machine (Himjin universal testing machine AGS-X1KN, Tianjin, China), which was equipped with a wedge-action tensile clamp. The tensile test was performed at a speed of 2 mm/min, and five specimens were tested and averaged to ensure the authenticity of the results. The stress–strain curve was used to calculate the tensile modulus.

#### 2.3.2. Scanning Electron Microscope (SEM)

Sections and fibers of tensile specimens with varying fiber contents were examined with scanning electron microscopy (Hitachi SU8010, Tokyo, Japan). Observations were made of the cross-section of the composite material at a voltage of 3.0 KV. The samples were coated with a thin layer of gold using sputtering equipment prior to SEM analysis.

#### 2.3.3. Fourier Transform Infrared Spectroscopy (FTIR)

The chemical composition of the specimens was analyzed using attenuated total reflection Fourier transform infrared spectroscopy (Thermo Scientific Nicolet iS20, Thermo Fisher Scientific, Waltham, MA, USA). An average of 32 scans was performed to obtain IR spectra in the range of 3100–450 cm^−1^ with a resolution of 4 cm^−1^.

#### 2.3.4. Differential Thermal Analysis (DSC)

The thermal properties of the specimens were tested using a differential thermal scanning calorimeter (Netzsch DSC 200 F3, Bavaria, Germany). The samples, weighing between 10 and 15 mg, were heated from room temperature to 250 °C at a heating rate of 10 °C/min in a nitrogen atmosphere with a flow rate of 50 mL/min, serving as a protective gas throughout the test.

## 3. Results and Discussion

### 3.1. Mechanical Test

**Figure 2:**
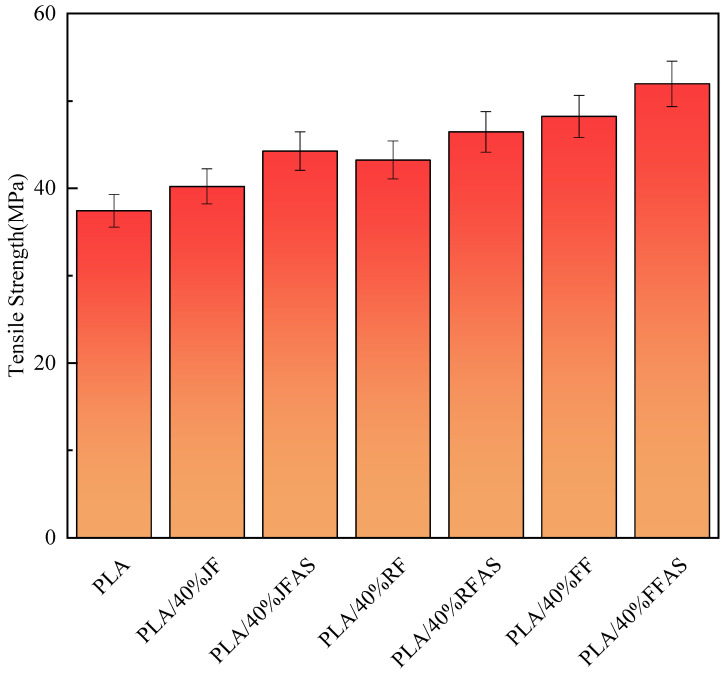
Tensile properties of PLA and the composites.

To investigate the effect of fiber pretreatment, we summarized the tensile strength of the three fiber-reinforced PLA composites before and after treatment at 40% content (as shown in Figure 2). For clarity, we named the specimens as follows: PLA/40% FFAS for 40% content of flax fiber-reinforced PLA composites treated with alkali and silane coupling agent together, JF for jute fiber, and RF for ramie fiber.

Figure 2 illustrates that the modification treatment leads to a significant enhancement in the tensile strength of the composite. Among the three fiber-reinforced PLA composites, the flax-reinforced PLA composites (FFRP) exhibit the highest tensile properties, followed by the RFPR and then the JFRP. The observed variation in the mechanical properties of the three fiber-reinforced PLA composites is consistent with the fundamental properties of the fibers, as presented in Table 1.

**Figure 3:**
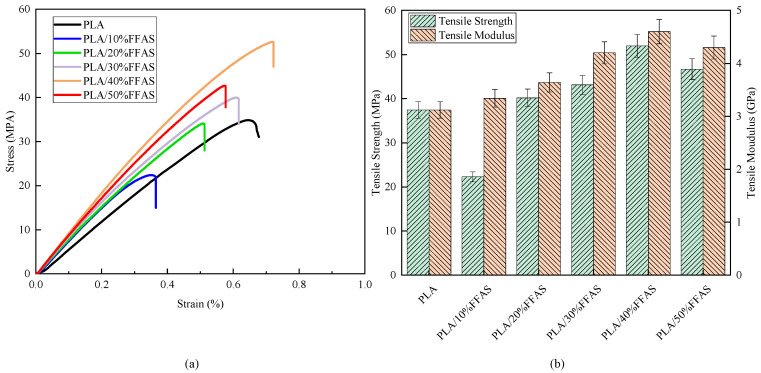
Mechanical properties of composites with different contents: (**a**) stress–strain curves; (**b**) tensile strength and tensile modulus.

Figure 3 summarizes the mechanical properties of FFRP with different contents. Comparing the tensile strength of pure PLA and FFRP with contents, when the fiber content is 10%, the tensile strength of the composites is lower than that of pure PLA. This phenomenon may be explained by the fact that at a lower fiber content, the load-bearing effect of the fibers may not be fully developed, and micro-defects may occur in the internal structure of the material. This can lead to reduced performance of the composite material. As the fiber content is increased further, the tensile properties of the composites continue to improve, with the maximum being achieved at 40% fiber content. The PLA matrix initially undergoes deformation under the influence of external forces. As the magnitude of the external forces increases, the stress is transferred from the matrix to the FF with greater strength, allowing the material to withstand higher external forces, resulting in an increase in the macroscopic tensile strength of the composite material [32]. However, when the fiber content exceeds 40%, the excess fibers cannot be uniformly dispersed in the PLA matrix, leading to the occurrence of agglomeration phenomena, which results in stress concentration and the initiation of micro-cracks within the material. Consequently, the macroscopic tensile strength of the composite material decreases [3,33].

### 3.2. SEM Results

**Figure 4:**
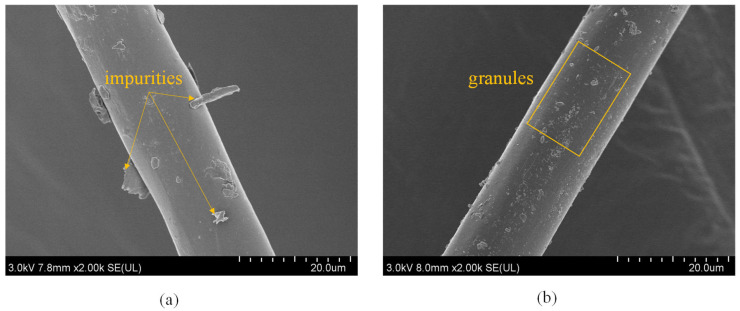
Microscopic morphology of fiber surface before and after treatment: (**a**) fiber before treatment; (**b**) fiber after alkali and silane coupling agent treatment.

To further illustrate the influence of the pretreatment process, we observed the microscopic morphology of the fibers before and after treatment with an alkali and silane coupling agent, as shown in Figure 4. As shown in Figure 4a, several obvious impurities adhere to the fiber surface, while the surface is smooth and shiny due to the waxy layer and silicide wrapped around the fiber. The surface of the treated fiber becomes uneven because the alkali soaking removes the waxy layer from the fiber surface. This results in a rougher fiber surface, which increases the specific surface area, improves the interfacial strength, and enhances the composite properties. This phenomenon has also been observed by Kamran et al. [34]. Numerous small particles can be observed on the fiber surface, as shown in the yellow rectangular box in Figure 4b, which are attributed to the reaction of the silane coupling agent with the polarized hydroxyl groups. This reaction leads to the formation of a layer of silane coupling agent coating on the fiber surface, which further improves the interfacial adhesion between the fiber and the PLA matrix [35].

**Figure 5:**
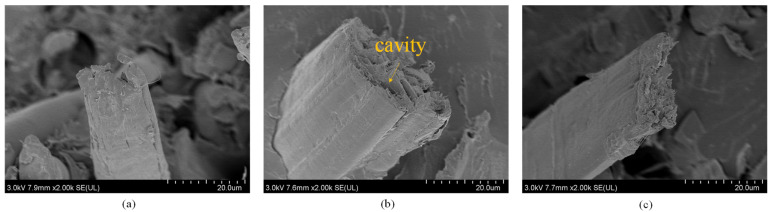
Three different fiber cross-sections: (**a**) ramie; (**b**) jute; (**c**) flax.

Figure 5 presents the cross-section of three distinct fibers. The microstructure of the three types of fibers exhibits significant differences, which may be attributed to the mechanical properties of the fibers (as presented in Table 1), and further influence the specific reinforcement effect. The weaker reinforcement of jute fiber in this study may be explained by the fact that jute fiber has more cavities in comparison to the other two fibers.

**Figure 6:**
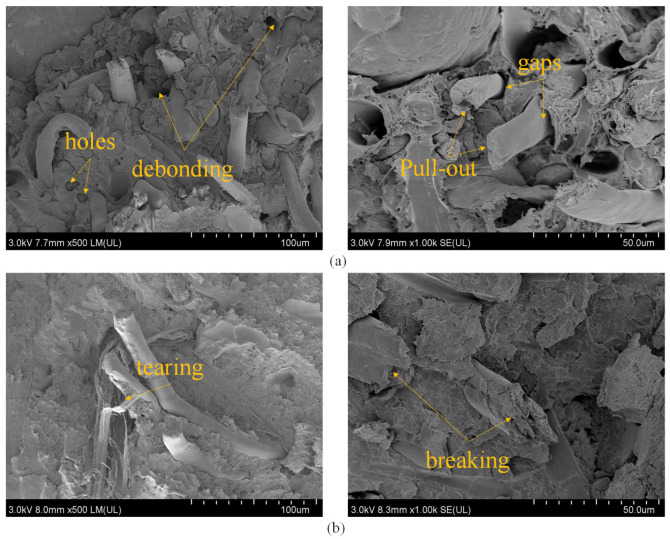
Comparison of sections before and after alkali and silane treatment of FF reinforced PLA composites: (**a**) before treatment; (**b**) after treatment.

The tensile fracture morphology of FFRP was observed to evaluate the fiber dispersion behavior in the PLA matrix and the interfacial properties between the fiber and matrix. As shown in Figure 6, the composite section without treatment displays more apparent holes and gaps between fibers and matrix. In contrast, the fracture surface of the composite treated with alkali and silane coupling agent exhibits fewer holes, and fiber breaking and tearing phenomena are observed. The rougher fiber surface caused by the alkali and silane coupling agent treatment leads to stronger bonding between the fibers and matrix, reducing the occurrence of fiber pull-out. Additionally, the fibers and matrix are more tightly bonded, with almost no gap, due to the silane coupling agent layer attached to the fiber surface after treatment. This layer is chemically bonded to the matrix, eliminating gaps between the fibers and the matrix. These observations support the enhancement of mechanical properties of the composite material after alkali and silane coupling agent treatment [36].

### 3.3. FTIR Results

**Figure 7:**
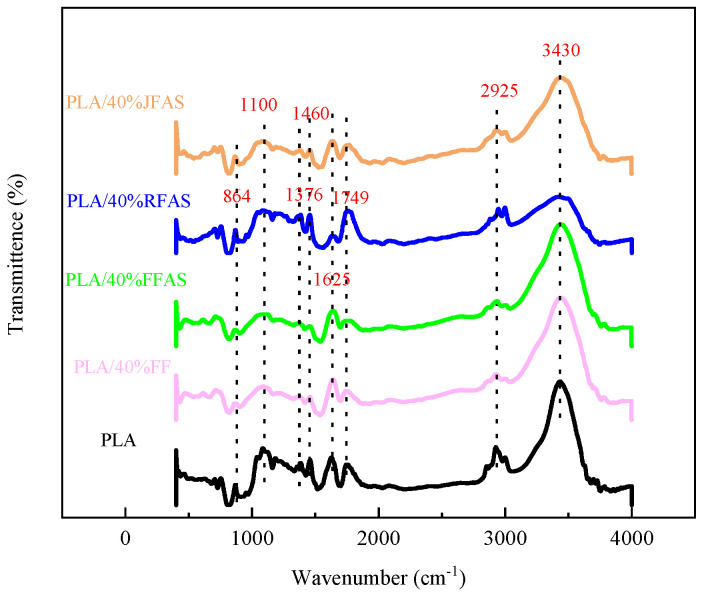
FTIR curves of the three fiber-reinforced PLAs after treatment, pure PLA, and FFRP before treatment.

The chemical structures of three types of composites, namely fiber-reinforced PLA composites treated with alkali and silane coupling agent, pure PLA, and untreated FFRP, were analyzed. The results presented in Figure 7 demonstrate that the peaks associated with the stretching of C-O, C=O, and C-H of lignin at 1100, 1749, and 2925 cm^−1^, respectively, were reduced after treatment with alkali and silane coupling agent. This reduction indicates the degradation of lignin [37]. The peaks observed at 1460 and 1625 cm^−1^, which are associated with the stretching of C=O and O-H of hemicellulose, respectively, were also reduced due to the partial removal of hemicellulose resulting from the treatment [38]. On the other hand, the absorbance of the peaks at 864 and 1376 cm^−1^, which are related to the C-H stretching of cellulose, did not significantly change after treatment. This result suggests that the structure of cellulose in the fibers was not affected by the alkali and silane treatment [38]. Furthermore, the positions of the peaks observed among the different fibers were similar, with variations only in their intensities. This finding suggests that the differences in the mechanical properties of the three fibers can be attributed to differences in their compositions [39].

In addition, it should be noted that silane treatment did not result in the loss of any peaks in the FTIR spectra. The removal of hemicellulose and lignin observed in Figure 7 is mainly attributed to the alkali treatment. It is worth noting that no peaks related to Si-O-Si and Si-C were observed in this study, most likely due to the low silane concentration used. Similar results were reported by Xiao et al. [37], who found it challenging to capture weak signals of Si-O-Si and Si-C peaks at low silane concentrations.

**Figure 8:**
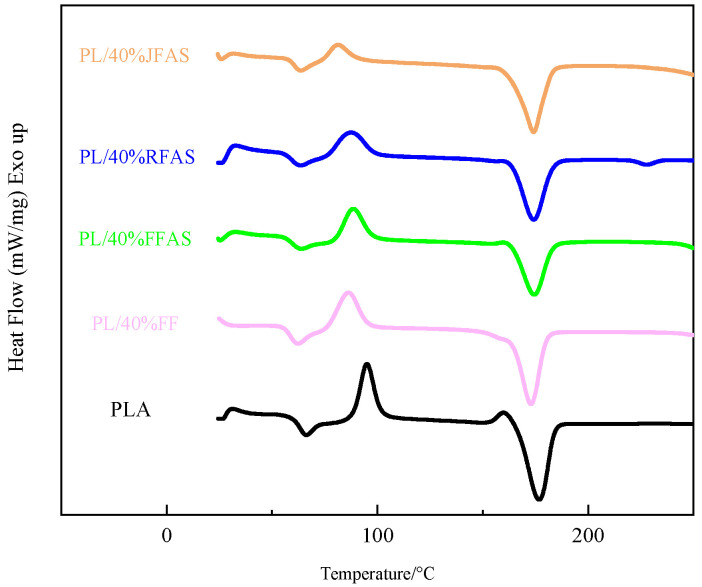
DSC curves of the three fiber-reinforced PLAs after treatment, pure PLA and FFRP before treatment.

The results of DSC tests for the three fiber-reinforced PLA composites after treatment and pure PLA and FFRP before treatment are shown in Figure 8 and summarized in Table 2. The glass transition temperature (T_g_), cold crystallization temperature (T_cc_), melting temperature (T_m_), and crystallinity (X_c_) of the composites were obtained.

Compared with PLA, T_g_ and T_cc_ were reduced in the reviewed PFRP, indicating that adding plant fibers to PLA reduced the melting and casting temperatures of the composites. According to the tensile performance data, the addition of plant fiber resulted in an increase in the tensile strength of composites, so it can be assumed that T_m_ and X_c_ also increased. However, DSC results showed the opposite trend, possibly due to the heterogeneous nucleation of fibers in the matrix [40]. All three temperatures of the alkali and silane coupling agent-treated composites were elevated compared with the untreated materials, and the increase in T_g_ was attributed to the fact that the silane coupling agent socking reduced the interfacial polarity between the fibers and PLA [41]. The elevated T_cc_ is caused by some chemical bonding between the silane coupling agent and the PLA chains, which restricts the PLA chains’ movement and makes the composite cold crystallization more difficult [41]. The increase in T_m_ and X_c_ indicates that the silane coupling agent forms a strong chemical bond between the fiber and PLA, reducing the number of free radicals in the composite [42]. This is consistent with the results obtained from DSC and tensile testing.

## 4. Finite Element Simulation

### 4.1. Theoretical Analyses

**Figure 9:**
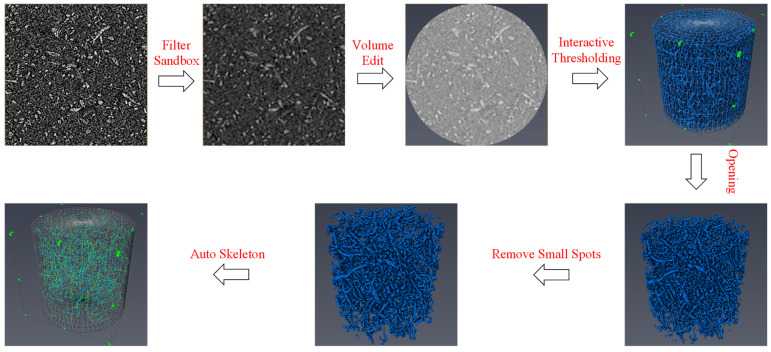
AVIZO software processing workflow.

The intermediate areas of the tensile specimens of the composites with different contents were subjected to X-ray computed tomography with a sample volume of 3 × 5 × 2 mm^2^, using a medium resolution of 3 um, in order to observe the real state of the fibers inside the composite. AVIZO 2019 software was used to reconstruct the fibers. Firstly, the original images were subjected to noise reduction. The model was cut to avoid the model being too large and causing operational difficulties (a circle with a diameter of 0.9 mm was extracted on the XY plane so that the threshold outside the circle was all reduced to zero, and the cylinder model with a diameter of 0.9 mm and a height of 0.9 mm was obtained by stretching along the *Z*-axis after the section was cut, to facilitate observation of the directional). To obtain more accurate features such as fiber orientation distribution, the slices were thresholded for segmentation (the intensity range of the threshold split is divided into fibers and matrix using 6350 as the split line). Fiber adhesion was found at this point, so the fiber surface’s small noise needs to be removed (take the ball type, size option 2, to remove the rough parts of the fiber surface, thus separating the fibers). After that, small pieces of islands were removed, as they were not well counted (by calculating the fiber content, the 3D model was selected to remove the islands with sizes less than 10 size). Finally, the extracted fibers were skeletonized to facilitate the counting of fiber length and orientation. The processing flowchart of AVIZO software is shown in Figure 9.

The analysis results showed that the composite fiber contents were 9.83%, 19.59%, 29.75%, 30.13%, 39.53%, and 49.96%, and the number of internal pores in the composite material is relatively low and does not significantly affect its performance, so we ignored it in this study, which was basically consistent with the experimental settings. However, the fibers are broken into small pieces through the twin-screw processing process so that the original length of the fibers is reduced, the average length of the fibers changes from the initial 3 mm to 0.2 mm, and the diameter is the original 8 um. The study also found that the distribution direction of fibers is related to the injection molding flow direction. The fibers are mainly distributed along the z direction (injection molding flow direction), so in this study, transversely isotropic constitutive was adopted to simulate the short fiber-reinforced composite materials fabricated by twin-screw injection molding [43,44].

Halpin and Tsai established a mathematical model for the elastic modulus of composite materials, which has been shown to be applicable to short fiber-reinforced composite materials [43,44]. Assuming that the fibers are completely aligned in the composite material, the transverse and longitudinal tensile strength and modulus can be expressed as Equations (1) and (2).

(1) $$E_{i}=E_{m}\left[\frac{1+\xi_{i}\eta_{i}V_{f}}{1-\eta_{i}V_{f}}\right]\;\;\;\eta_{i}=\frac{\frac{E_{f}}{E_{m}}-1}{\frac{E_{f}}{E_{m}}+\zeta_{i}}\;\;\zeta_{1}=\frac{2L}{D}\;\;\zeta_{2}=2\;\left(i=1,2\right)$$

(2) $$\sigma_{i}=\sigma_{m}\left[\frac{1+\xi_{i}\eta_{i}V_{f}}{1-\eta_{i}V_{f}}\right]\;\;\;\eta_{i}=\frac{\frac{\sigma_{f}}{\sigma_{m}}-1}{\frac{\sigma_{f}}{\sigma_{m}}+\zeta_{i}}\;\;\zeta_{1}=\frac{2L}{D}\;\;\zeta_{2}=2\;\left(i=1,2\right)$$

where $E_{f}$ and $E_{m}$ represent the moduli of the fiber and matrix, $\sigma_{f}$ and $\sigma_{m}$ represent the tensile strengths of the fiber and matrix, L represents the average fiber length, D represents the fiber diameter, $V_{f}$ represents the volume fraction of fiber, the subscripts 1 and 2 represent the transverse and longitudinal directions, and ζ is a parameter affected by the fiber filling arrangement and geometry.

As the fiber content increases, the fibers are more likely to agglomerate and produce agglomeration effects affecting the composite properties, and in order to reflect the negative effects of fiber agglomeration, Facca et al. [45] proposed a fiber agglomeration factor function Χ that is related to the fiber content. They defined Χ as the ratio of the fiber surface area available for stress transfer to the total fiber surface area. The formula is as follows:

(3) $$Χ=1-\frac{V_{f}}{V_{fmax}}$$

where $V_{fmax}$ represents the maximum volume fraction that can be added to the material without producing significant defects. Later, Yang et al. [44] corrected the fiber volume fraction using a fiber agglomeration factor as follows:

(4) $$V_{fc}=XV_{f}$$

**Figure 10:**
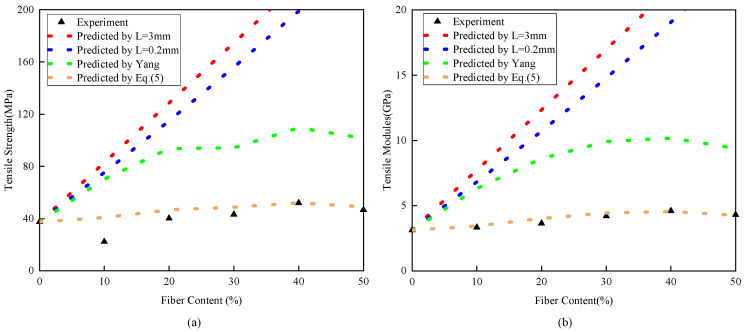
Experimental results and estimations of theoretical calculations: (**a**) tensile strength; (**b**) tensile modules.

By using the above method, the properties of the composite material processed in this study were predicted, and the results are shown in Figure 10, which shows significant deviations from the experimental results. By observing the reconstructed fibers using AVIZO software, it was found that the twin-screw process would change the fiber length and shape, and some fibers would be block-shaped. Moreover, this phenomenon becomes more pronounced with increasing fiber content. Based on this phenomenon, the fiber agglomeration factor function was introduced to further correct ζ, and the formula is as follows:

(5) $$\zeta_{c}=Χ\zeta$$

Similarly, the results are presented in Figure 10, which shows that the theoretical formula corrected by Equation (5) significantly reduces the error between the prediction and experimental results. It is worth noting that the experimental results and theoretical formula predictions of the composite material with 10% fiber content have a large difference. This is because the formula is mainly used to reflect the forward strengthening effect of fibers on the matrix, while the 10% fiber content increases the internal defects of the material, leading to a decrease in performance. This indicates that this content should be avoided in actual processing. In order to achieve the purpose of strengthening PLA, a fiber content of at least 20% should be selected.

### 4.2. Numerical Simulation Methods

Through the theoretical formula analysis, the accurate transverse isotropic elastic parameter and tensile strength parameter of the material can be obtained. Considering that the composite material may be under different loading conditions in actual service, in order to simulate material failure and predict performance under complex stress conditions, a numerical simulation method was developed by combining the material constitutive model based on the theoretical analysis with the UMAT subroutine.

The UMAT user-defined subroutine has been implemented in ABAQUS/Standard to simulate the failure behavior of these composites. The Tsai–Wu criterion is considered to provide the most comprehensive description of composite damage compared to other criteria. It is possible to simplify all other criteria to obtain this criterion based on specific loading stress conditions. Consequently, the fitted curves obtained using this criterion show better agreement with experimental results and can more accurately predict the strength of the composites. The Tsai–Wu criterion is presented in Equation (6).

(6) $$F_{1}\sigma_{1}+F_{2}\sigma_{2}+F_{3}\sigma_{3}+F_{11}\sigma_{1}^{2}+F_{22}\sigma_{2}^{2}+F_{33}\sigma_{3}^{2}+2F_{12}\sigma_{1}\sigma_{2}\;+2F_{23}\sigma_{2}\sigma_{3}+2F_{31}\sigma_{3}\sigma_{1}+F_{44}\tau_{23}^{2}+F_{55}\tau_{31}^{2}+F_{66}\tau_{12}^{2}=1$$

where the coefficients of the tensor $F_{i}$ and $F_{ij}$ are determined by X_T_, X_C_, Y_T,_ Y_C_, Z_T_, Z_C_, S_23_, S_31_, and S_12_, as shown in Equation (7). The subscripts T and C denote the tensile and compressive states, respectively, and S indicates the shear state.

(7) $$F_{1}=\frac{1}{X_{T}}-\frac{1}{X_{C}},F_{2}=\frac{1}{Y_{T}}-\frac{1}{Y_{C}},F_{3}=\frac{1}{Z_{T}}-\frac{1}{Z_{C}},\;F_{11}=\frac{1}{X_{T}X_{C}},F_{22}=\frac{1}{Y_{T}Y_{C}},F_{33}=\frac{1}{Z_{T}Z_{C}},\;F_{44}=\frac{1}{S_{23}^{2}},F_{55}=\frac{1}{S_{31}^{2}},F_{66}=\frac{1}{S_{12}^{2}},\;F_{12}=-\frac{1}{2\sqrt{X_{T}X_{C}Y_{T}Y_{C}}}\;F_{23}=-\frac{1}{2\sqrt{Y_{T}Y_{C}Z_{T}Z_{C}}}\;F_{31}=-\frac{1}{2\sqrt{X_{T}X_{C}Z_{T}Z_{C}}}$$

**Figure 11:**
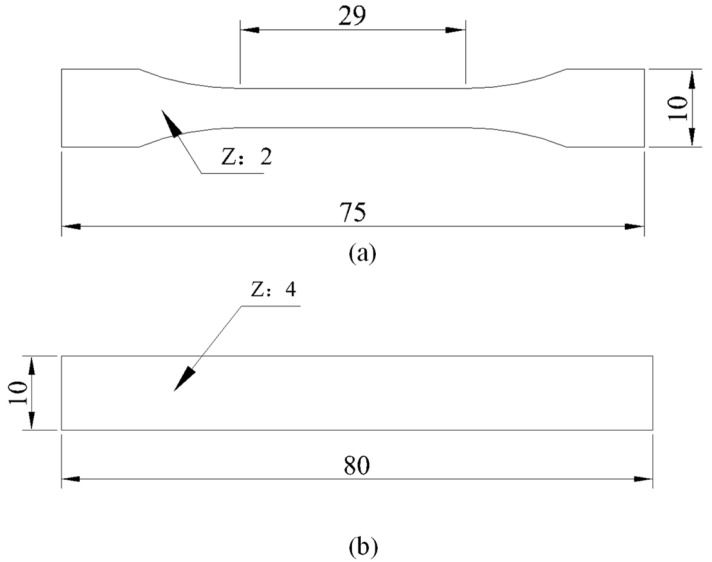
Specimen size (mm): (**a**) dumbbell tensile specimens; (**b**) three-point bending specimens.

**Figure 12:**
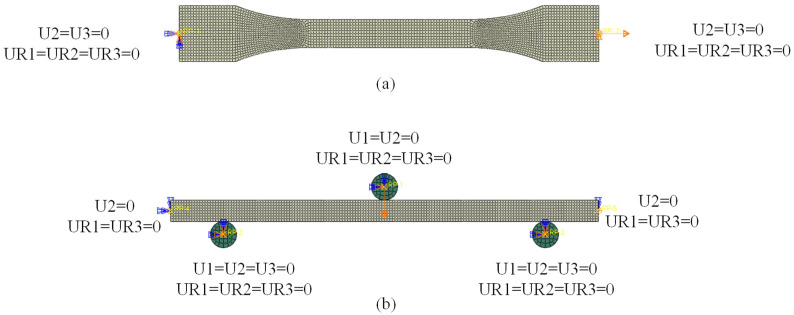
Finite element model: (**a**) dumbbell tensile specimen; (**b**) three-point bending specimen.

The 3D finite element models of the dumbbell tensile specimen and three-point bending specimen were established using the ABAQUS 2021 software for numerical simulation analysis. The 3D models of the dumbbell tensile specimen and three-point bending specimen were established in accordance with the standards of ASTM D638 and ISO178, respectively, as depicted in Figure 11. The 3D finite element models (FEM) of the dumbbell tensile specimen and the three-point bending specimen were established using ABAQUS and are illustrated in Figure 12. Both specimens were modeled using 3D stress elements (C3D8R) with swept meshing using the neutral axis algorithm. The mesh type used was hexahedral, and a global seeding was used. Approximate global sizes ranging from 0.3 to 0.05 were selected to test mesh convergence. It was observed that the results were no longer affected by the mesh when the element size was smaller than 0.1. Therefore, a global size of 0.1 was chosen. The static general analysis step was used, with geometric nonlinearity enabled to prevent results from failing to converge due to large displacements. One end of the dumbbell tensile specimen was fixed using an encastre constraint to restrict all translational and rotational degrees of freedom, while the other end was applied with translational displacement along the x direction to restrict the remaining degrees of freedom. The three-point bending specimen included an indenter and two brackets that were face-to-face constrained to the corresponding surfaces. The two supports were fixed using an encastre constraint; the indenter displacement in the z direction and limited degrees of freedom in the remaining direction. Both the left and right ends were constrained to move smoothly in the x and z directions and to rotate in the y direction. The ABAQUS/Explicit module was used for analysis, and the geometric nonlinearity of materials was considered.

### 4.3. Analysis of Simulation Results

**Figure 13:**
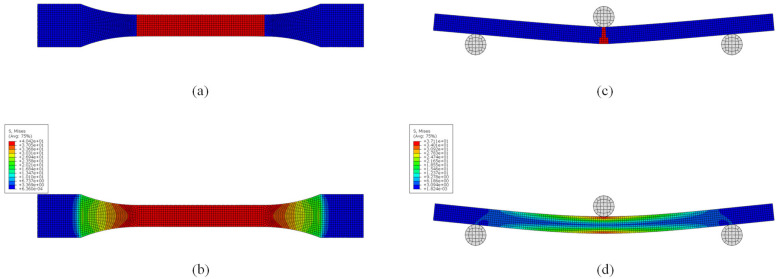
Finite element simulation results: (**a**) tensile failure point; (**b**) stress cloud before tensile failure; (**c**) bending failure point; (**d**) stress cloud before bending failure.

To validate the accuracy of the proposed model, PLA materials were chosen, and 3D FEMs of the tensile and three-point bending specimens were established in ABAQUS/Standard. The simulation parameters are presented in Table 3, and the simulated and experimental tensile and bending results of PLA are depicted in Figure 13. The load–displacement comparison of the simulation and experimental results for tensile and three-point bending are summarized in Table 4. The simulation results are consistent with the failure positions in the experiments (the tensile test failed from the middle section, while the bending test failed from the middle of the bottom surface). The strength results are similar, and the displacement errors are small, which verifies the feasibility of the proposed model.

**Figure 14:**
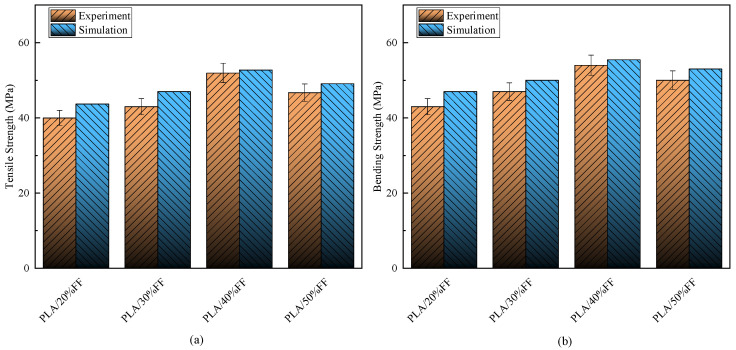
Comparison of experimental and simulation: (**a**) tensile strength; (**b**) bending strength.

To verify the accuracy of high-content plant fiber composite materials, different content composite materials were further simulated. The 2-directional strength and modulus were calculated using the theoretical function corrected by Equation (5). The theoretical formula data were used to simulate the model, and the simulation results were compared with the experimental results, as shown in Figure 14. The error between the simulation results and the experimental data was within 10%, which proves that the model has high accuracy.

## 5. Conclusions

In this study, we investigated the mechanical properties of short PFRP with different fiber contents using three fibers (flax, ramie, and jute) and a twin-screw extrusion process. The composites were characterized by various techniques, including mechanical property testing, SEM, FTIR, and DSC. Additionally, we established a rapid mechanical properties prediction method for short PFRP based on the content degradation factor through simulation. Our findings suggest that:

(1) Alkali treatment can effectively remove impurities, pectin, and wax from the surface of the fiber, consequently enhancing the roughness of the fiber surface. Silane coupling agent treatment forms chemical bonds between the fibers and the PLA. The combined effects of alkali and silane treatments improve the interfacial connectivity, thus enhancing the mechanical properties of the composite materials.

(2) Flax fiber showed the highest effectiveness in reinforcing PLA when compared to ramie and jute fibers. The mechanical properties and load-bearing capacity of the composite increased within a specific content range as the fiber content increased, with the best performance observed at 40% content. However, with further increases in fiber content, the fiber distribution became uneven, leading to fiber agglomeration and a reduction in composite performance due to stress concentration and micro defects. Additionally, an interesting phenomenon was discovered in the study. At low fiber content (10%), the performance of the composite is lower than that of pure PLA. This may be attributed to the incomplete transfer and load-bearing capacity of fibers at low fiber content, and they may even contribute to internal defects within the material.

(3) The fiber was reconstructed using Micro-CT technology, and it was found that the twin-screw process alters the shape and length of the fiber, and the fiber is distributed mainly towards the direction of injection molding flow. By incorporating the fiber agglomeration factor function, the Halpin–Tsai criterion was corrected. Transversely isotropic numerical predictions were effectively integrated with finite element analysis to accurately predict the mechanical properties of short plant fiber-reinforced composites with varying fiber contents. The simulation results were in strong agreement with the experimental results.

## Figures and Tables

**Table 1:** Mechanical properties of three fibers and PLA.

Material	$\rho \left(g/cm^{3}\right)$	$S\left(MPa\right)$	$E\left(GPa\right)$	$\varepsilon \left(\%\right)$
Flax Fiber	1.5	500	50	2
Jute Fiber	1.46	400	40	1.8
Ramie Fiber	1.5	460	45	2
PLA	1.24	40	3	4

**Table 2:** Glass transition temperature (T_g_), cold crystallization temperature (T_cc_), and melting temperature (T_m_).

Sample	T_g_(°C)	T_cc_(°C)	T_m_(°C)	X_c_(%)
PLA	61.62	95.03	176.76	50.11
PLA/40%FF	59.53	86.25	172.84	42.37
PLA/40%FFAS	60.06	88.53	174.47	44.34
PLA/40%RFAS	59.87	87.48	174.16	43.21
PLA/40%JFAS	58.94	81.29	174.17	42.53

**Table 3:** PLA material simulation parameters.

Parameter Type	Numerical Value
Elastic modulus (GPa)	3.1
Poisson’s ratio	0.3
Tensile strength (MPa)	37.43
Compressive strength (MPa)	42.21
Shear strength (MPa)	20.79

**Table 4:** Load and displacement comparison between test and simulation.

		Experiment	Simulation	Error Range (%)
Tensile	Load (N)	348.59	360.42	3
	Displacement (mm)	0.48	0.50	4
Bend	Load (N)	66.34	68.72	4
	Displacement (mm)	2.06	2.12	3

## Data Availability

If data is needed, please contact the author.
